# Uncovering Novel Protein Partners of Inducible Nitric Oxide Synthase in Human Testis

**DOI:** 10.3390/biom14040388

**Published:** 2024-03-24

**Authors:** Karthik S. Prabhakara, Kavya Ganapathy, Kazi N. Islam, Hiran M. Thyagarajan, Kirti K. Tiwari, Ramya L. Parimi, Mohammad B. Rashid

**Affiliations:** 1 Department of Biology and Biotechnology, College of Science and Engineering, University of Houston-Clear Lake, 2700 Bay Area Blvd., Houston, TX 77058-1098, USA; 2 Department of Agricultural Research and Development Program, Central State University, 1400 Brush Row Road, Wilberforce, OH 45384, USA

**Keywords:** male infertility, nitric oxide synthase, protein–protein interaction, yeast two-hybrid assay, co-immunoprecipitation

## Abstract

Peroxidative damage to human spermatozoa has been shown to be the primary cause of male infertility. The possible role of nitric oxide (NO) in affecting sperm motility, capacitation, and acrosome reaction has been reported, too. The overproduction of NO by the enzyme inducible nitric oxide synthase (iNOS) could be responsible as it has been implicated in the pathogenesis of many diseases. There have been many studies on regulating iNOS function in various tissues, especially by protein–protein interaction; however, no study has looked for iNOS-interacting proteins in the human testis. Here, we have reported the identification of two proteins that interact with iNOS. We initially undertook a popular yeast two-hybrid assay to screen a human testis cDNA library in yeast using an iNOS-peptide fragment (amino acids 181–335) as bait. We verified our data using the mammalian chemiluminescent co-IP method; first, employing the same peptide and, then, a full-length protein co-expressed in HEK293 cells in addition to the candidate protein. In both cases, these two protein partners of iNOS were revealed: (a) sperm acrosome-associated 7 protein and (b) retinoblastoma tumor-suppressor binding protein.

## 1. Introduction

Male infertility can result from inflammation of the male reproductive tract [1,2,3]. Many studies have shown that oxidative stress invalidated spermatozoa, including free radical nitric oxide (NO) [4]. NO is a highly reactive gaseous molecule produced by the three enzymatic isoforms of nitric oxide synthase (NOS). Out of these, endothelial NOS (eNOS) and neuronal NOS (nNOS) function constitutively, producing the basal level of NO in a calcium-dependent manner; however, due to sustained stability of inducible NOS (iNOS) functioning at a very basal calcium level, a bolus of NO is produced causing a tumultuous change in the local subcellular environment [5]. These enzymes share a remarkable structural homology, with the most contrasting difference in the amino-terminal regions. Three structural domains common to all are: (a) an oxygenase domain that is amino-terminal and binds to L-arginine, BH4, and heme; (b) a carboxy-terminal reductase domain that binds to FAD, FMN, and NADPH; and (c) a calmodulin-binding domain that connects these two domains. A unique testes-specific nNOS, TnNOS, has also been reported in Leydig cells that can produce NO [6]. It is implied that a high level of NO produced in the testis would impair sperm motility; this is supported by the finding that Leydig cells under LPS-mediated inflammation could produce high local NO, thus contributing to male infertility [3]. Overproduction of nitric oxide by iNOS has also been linked to renal damage due to peroxidative injury [7]. Also, NO has been reported lately in the maturation of follicular development in female rats [8]. Infertility in both males and females was found to be concurrent with urinary tract infection, suggesting a role of local NO synthesis by iNOS. Previously, anticipation was similarly expressed after observation of the number of immotile spermatozoa in the semen collected from different individuals which had a linear relationship to the amount of NO in the tissue [9].

Cell signaling plays a vital role in *iNOS* gene expression and may even modulate its subcellular localization or compartmentalization [10]. The promoter region exhibits intricate control over its expression, making it a tightly regulated process [11]. Subcellular localization of enzymes in the cell can limit or enhance its activity. Localization of iNOS to aggresomes present at the microtubule organizing center (MTOC) during stress has been reported [12]. NO synthesis can be regulated at various levels, e.g., at the co-transcriptional level, post-transcriptionally by alternative splicing, post-translational modifications, protein–protein interactions, and by subcellular localization of the NOS enzymes [13,14].

Protein–protein interaction plays a vital role in the functions and regulations of many enzymes, and evidence indicates that NOSs may have similar interactions. Many proteins interacting with various NOS isoforms have been identified [1]. In addition to the general affinity that the calcium-binding protein calmodulin has for all NOSs, several proteins that differentially interact with NOSs have been identified. Studies have shown that in addition to binding to CaM, the iNOS protein interacts with several other proteins, e.g., Kalirin, NAP110, etc. The Kalirin protein is involved in neuronal growth and maintenance in adult human brains and can inhibit iNOS function [15,16]. The NAP110 is an NOS-associated protein that binds to the iNOS oxygenase domain in murine macrophage cells [17] and prevents iNOS dimerization [15]. Other examples of reported iNOS-interacting proteins are Rho GTPase Rac2 [18] and hsp90 [19]. This means hsp90 also interacts with all the NOS isoforms.

Further bolstering the protein–protein interaction of all NOSs, recent research with site-directed mutagenesis has identified critical amino-terminal amino acid residues in human iNOS that are important for dimerization [20]. Since the amino acid sequence of three NOSs is different in its amino-terminal portions, it is believed that additional proteins may be interacting with the NOSs.

We have initially undertaken a yeast two-hybrid assay (Y2H) system from TaKaRa Bio USA, Inc. This Matchmaker^®^ gold Y2H system is based on the novel technique invented by Fields and Song in 1989 [21]. We screened a human testis cDNA library in yeast to identify candidate proteins that may interact with the unique iNOS bait fragment. Then, we opted for confirmation of these putative interactions with a mammalian Matchmaker^®^ Co-IP system (TaKaRa Clontech, Inc., PT3929-1).

## 2. Materials and Methods

### 2.1. Materials Used in This Study

An In-Fusion^®^ HD EcoDry™ cloning kit (cat. #638913), a Matchmaker^®^ gold yeast two-hybrid system (cat. #630489), a Mate & Plate human testis cDNA library (cat. #630470), a CalPhos™ Mammalian Transfection Kit^®^ (cat. #631312), a Matchmaker^®^ chemiluminescent Co-IP vector set (cat. #630458), and a ProLabel detection kit II (cat. #631629) were purchased from Takara Bio USA, Inc. Additional pAmCyan1-C1 vector was also purchased from the same manufacturer (cat. #632441) for immunofluorescence studies. Minimal synthetic defined (SD) bases and dropout (DO) supplements for confirming auxotrophic phenotypes and selecting yeast transformants were purchased from Takara Bio USA, Inc. The oligonucleotides were ordered from Integrated DNA Technologies, Inc., USA. RedTaq^®^ ready mix (MilliporeSigma Inc., Burlington, MA, USA cat. #R2523) was used to amplify iNOS DNA fragments. The restriction enzymes and T4 DNA ligase were purchased from New England Biolabs, Ipswich, MA, USA, Inc. Bacterial and yeast culture media were prepared following standard protocols. The mammalian cell line was human embryonic kidney HEK293 (ATTC^®^ CRL-1573™). The primary antibodies used were anti-NOS2 (H-174, cat. #SC8310), and anti-SPACA7 (C-16, cat. #SC84019) from Santa Cruz Biotechnology Inc., Dallas, TX, USA. Also, the anti-RbBp4 clone 6G5.1 (EMD Millipore, Inc., USA, cat. #MABE221) and anti-cMyc (Takara Bio USA, Inc.) were used. For RT-PCR, we obtained the siRNA reagent system (#sc-45064) and siRNA vectors specific to the proteins SPACA7 (cat. #sc-105143) and RbBp4 (cat. #sc-37962) from Santa Cruz Biotechnology USA, Inc. An RNeasy mini kit (cat. #74104) was from Qiagen Inc., Hilden, Germany. The ProLong^®^ diamond antifade mountant with DAPI (cat. #P36966) was purchased from Life Technologies Inc., Carlsbad, CA, USA. A high-capacity cDNA archive kit (cat. #4368814) was obtained from Applied Biosystems Inc., Waltham, MA, USA. Fluorophore-conjugated secondary antibodies (cat. #DI-1794 2014) were bought from Vector Laboratories Inc., Newark, CA, USA.

### 2.2. Yeast Two-Hybrid Assay

The Y2H was carried out according to the manufacturer’s instructions for the Matchmaker^®^ gold yeast two-hybrid system. This technique exploits a new yeast strain that uses four separate reporter gene expressions to select for protein interaction, thus eliminating many false positives. A PCR-amplified human iNOS DNA fragment was generated, serving as bait in the assay. The Matchmaker^®^ gold yeast two-hybrid system has the bait-cloning vector pGBKT7-DNA-BD and positive controls as pGBKT7-53 and pGADT7-T. The kit also contains *S. cerevisiae* Y2HGold™ and *S. cerevisiae* Y187 strains for yeast-mating purposes. We employed a human testis cDNA library premade in a pGADT7-AD vector and transformed into Y187 cells.

### 2.3. Subcloning of iNOS Fragments into pGBKT7 Vector Plasmid

A PCR-amplified human iNOS DNA fragment was generated, serving as bait in the assay. The iNOS cDNA was initially obtained as a gift from Prof. N. Tony Eissa of the Baylor College of Medicine, Houston, TX, USA. The forward and reverse primers (see below) were designed to amplify the region of iNOS-encoding amino acids 181–335. The forward primer contained NdeI and the reverse primer contained SalI cutting sites (in lowercase) for directional cloning of the insert into a similarly cut pGBKT7 plasmid; this allowed the fusion of the iNOS sequence (encoding amino acids 181–335) in frame with the GAL4 DNA-binding domain. Ligation of DNA ends was performed by T4 DNA ligase following standard ligation protocol.

NOS(181)FWD: 5′-AGGAGGACCTGcatatgCTGACGGGAGATGAGCTCATC-3′

NOS(335)REV: 5′-GCCGCTGCAGgtcgacTTTGGGATGTTCCATGGC-3′

### 2.4. Yeast Transformation and Mating

A small-scale lithium acetate transformation of different yeast strains was performed. Yeast mating was performed to introduce plasmids with different markers into the same host cell, as instructed by the manufacturer (Takara Bio USA, Inc., #PT3024). The control yeast mating was also performed for the plasmids containing p53 and T-antigen that had been transformed into *S. cerevisiae* strains Y187 and AH109, respectively. For selection, appropriate single-dropout media (SD/-Leu and SD/-Trp), double-dropout media (SD/-Leu/-Trp), and quadruple-dropout media (SD/-Leu/-Trp/-His/-Ade) were used. DDO/X/A indicates double-dropout media without leucine and tryptophan, including X-alpha Gal and Aureobasidin A. The colonies that appeared on the DDO/X/A plates were reconfirmed by streaking onto quadruple-dropout media QDO/X/A plates to look for the functioning of all four reporter gene expressions in the presence of X-alpha Gal and Aureobasidin A.

### 2.5. Construction of Mammalian AcGFP1-Bait and pProLabel-Prey Plasmids Using In-Fusion^®^ Cloning

The In-Fusion^®^ cloning experiments were set up according to the manufacturer’s protocol. Specific primers were designed according to the user manual of the In-Fusion^®^ HD EcoDry™ cloning kit. The mammalian vectors, pAcGFP1-C and pProLabel-C, were double digested using HindIII/*Sal*I-HF^®^ and BamHI-HF^®^/SalI-HF^®^ enzymes, respectively. The same iNOS bait DNA was inserted into the pAcGFP1-C vector and the human cDNA testis library preys into the pProLabel-C plasmid vector.

### 2.6. Transfection of HEK293 Cells

For transfection with a single or double plasmid(s), the CalPhos™ Mammalian Transfection Kit^®^ was used. Expression of green fluorescent protein (GFP)-fused bait protein was determined by fluorescence imaging using a Nikon Eclipse 80i advanced research microscope under the FITC filter at 20× magnification.

### 2.7. Co-Immunoprecipitation and ProLabel Assay

Matchmaker^®^ Chemiluminescent Co-IP Assay Kit II^®^ protocol was followed to immunoprecipitate GFP-tagged iNOS fragment; Living Colors^®^ full-length GFP polyclonal antibody was used in the Co-IP. To precipitate down the antibodies, Pierce© protein A/G plus agarose beads were utilized. This method utilizes a fluorescent AcGFP1 tag and the enzymatic ProLabel^®^ reporter for chemiluminescent detection of physical interactions between two proteins in the mammalian cells. Briefly, we used the following protocol: the diluted lysate (250 µg) was incubated with 1 µL of Living Colors^®^ full-length GFP polyclonal antibody. Pierce^®^ protein A/G plus agarose beads were then added to this and mixed overnight at 4 °C. The beads were washed, resuspended in lysis/complementation buffer, and transferred to the OptiPlate^®^ 96-well plate. The substrate mix was added to each well and the luminescent activity for each sample was measured at 0, 10, 20, 30, 40, 50, and 60 min intervals using the TopCount^®^ NTX microplate scintillation and luminescence counter (Packard BioScience Co., Meriden, CT, USA). The experiment was repeated at least three times to verify the reproducibility.

### 2.8. Real-Time PCR

Total RNA was isolated from the transfected cells using an RNeasy^®^ mini kit. The cDNA was synthesized using a high-capacity cDNA archive kit. The cDNA thus prepared was used to quantify the mRNA expression using a StepOnePlus™ real-time PCR system (Life Technologies Inc., Carlsbad, CA, USA, cat. #4376600) and analyzed by using the comparative CT method. We used a fast SYBR^®^ green master mix in the assay.

### 2.9. Fluorescence Imaging of Co-Transfected HEK293 Cells

Cloning in pAcGFP-C and pAmCyan1-C1 plasmids results in the expression of proteins tagged with GFP and CFP (cyan fluorescent protein), respectively. Therefore, the expression of the bait and prey proteins in the human embryonic kidney cell line (HEK293) could be imaged using an E80i Nikon fluorescence microscope under the FITC filter as both GFP and CFP can be viewed at 515/30 nm (median wavelength/bandwidth) [22]. Stably transfected cells were seeded on a coverslip and were subjected to incubation in the dark with ProLong^®^ diamond antifade mountant with DAPI. We then sealed the slides around the coverslips and viewed them under a fluorescence microscope using the DAPI and FITC filters. For immunostaining, we fixed transfected cells with 4% paraformaldehyde on the coverslips, treated them with blocking buffer (1X PBS, 2.5% normal horse serum), and then incubated them with the primary antibody for 12 to 16 h. The respective fluorophore-conjugated Vecta-Fluor™ secondary antibodies were added and incubated in the dark for 30 min. After washing, each coverslip was individually mounted on pre-cleaned glass slides containing a drop of the antifade mountant with DAPI. The slides were viewed under a fluorescence microscope using the DAPI, FITC, and TRITC filters.

## 3. Results

### 3.1. Confirmation of Expression of Bait Protein in Yeast Cells by Western Blotting

**Figure 1:**
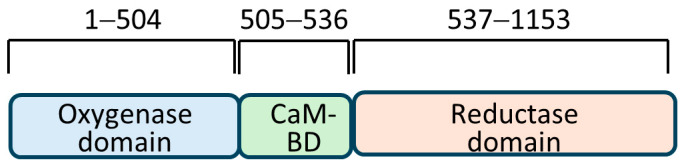
Three domains of human inducible nitric oxide synthase with corresponding amino acid residues are shown above (not according to scale). CaM-BD = calmodulin-binding domain.

**Figure 2:**
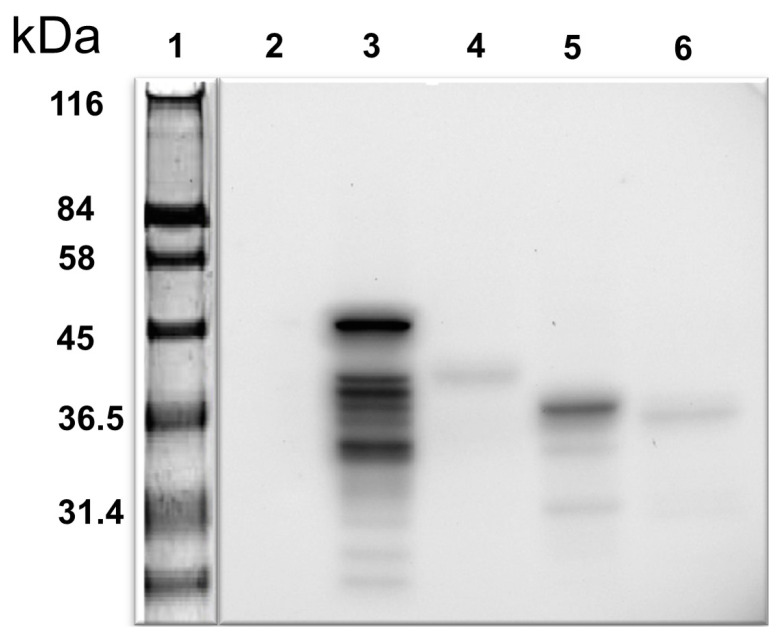
Western blot of iNOS bait in yeast-transformants using anti-cMyc antibody. Lane 1: pre-stained protein marker; Lane 2: negative control—untransformed yeast lysate; Lane 3: positive control—yeast transformed with pGBKT7-53; Lanes 4 and 6: other iNOS protein fragments faintly displayed (not used in this study); Lane 5: iNOS peptide fragments from 181 to 335aa used in this study. cMyc is a fused tag.

The bait employed in the Y2H assay was obtained by successfully amplifying a 465 bp DNA fragment encoding the amino acid residues 181–335 of the human iNOS. The bait belonged to the oxygenase domain of the iNOS (Figure 1). DNA inserted into the bait-cloning vector pGBKT7 was confirmed by DNA sequencing. The iNOS protein fragment expression in yeast (Y2HGold™) cells was tested after transformation with pGBKT7-NOS bait plasmid. Cell lysates were prepared as per the protocol and a standard Western blotting experiment was performed employing a primary antibody to the c-Myc epitope (Figure 2). We identified a protein band corresponding to around 36 kDa (Lane 5, Figure 2), the protein’s expected size. Lysate made from Y2HGold™ transformed with pGBKT7-53 was used as a positive control.

### 3.2. Qualitative Analysis of Bait-and-Prey Interaction

In the Y2H system, two yeast strains of opposite mating types are exploited. The budding Y2HGold™ bait cells were mated with Y187 cells transformed with material from the human testis cDNA library. Successfully mated yeast cells were screened on selective DDO/X/A plates (Leu and Trp-dropout plates plus X-alpha Gal and Aureobasidin A). We observed several blue colonies indicative of reporter gene expression from putative protein–protein interaction. For further verification, we restreaked these blue-colored colonies onto QDO/X/A (additional Ade and His dropout) plates. Yeast clones that grew on QDO plates were selected for plasmid DNA isolation. The isolated plasmids were amplified by propagation through competent *E. coli* cells. The human testis cDNA containing plasmids were retransformed into Y187 cells and, again, allowed to mate with Y2HGold™/NOS cells. The ones that showed consistent results as before were picked for analysis by DNA sequencing. Nucleotide sequences of two proteins, Spaca7 and RbBP4, were obtained.

### 3.3. Co-Immunoprecipitation and ProLabel Assay to Confirm Protein–Protein Interaction

**Figure 3:**
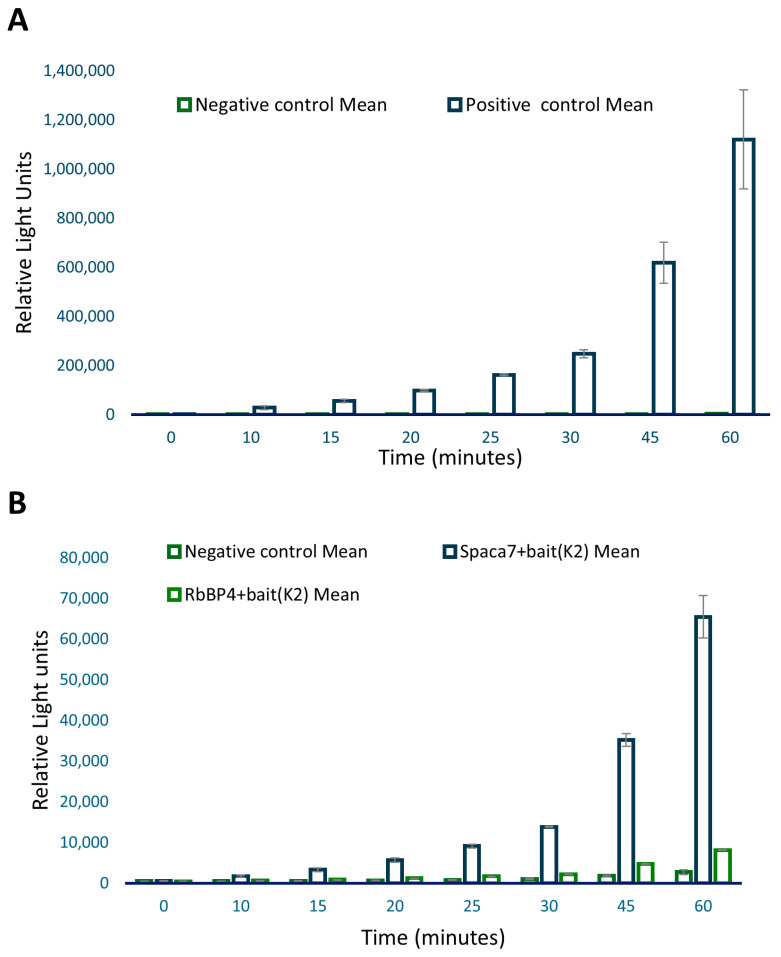
ProLabel assay after co-immunoprecipitation. Panel (**A**) shows the PL assay with positive control p53 and its interacting partner T antigen. Panel (**B**) shows the PL assay with candidate proteins, Spaca7 and RbBP4. The negative control included AcGFP1-Lam plus ProLabel-SV40 T.

Each testis-specific cDNA identified by Y2H was cloned into the pProLabel-C vector to incorporate the ProLabel (PL) tag, enabling chemiluminescent detection. The mammalian vectors expressing AcGFP-NOS and PL-prey fusion proteins were co-transfected into HEK293 cells. Co-immunoprecipitation was performed as detailed in the Materials and Methods section. Proteins obtained by Co-IP were then tested for the ability of the PL tag to combine with an enzyme acceptor provided in the kit and complete an ‘active enzyme’ that cleaves a chemiluminescent substrate, generating a chemiluminescent signal. The p53 and T-antigen pair were positive controls to validate the other interactions. Co-IP and ProLabel assay were performed as described in the Materials and Methods section. The luminescent activity was measured at 0, 10, 15, 20, 25, 30, 45, and 60 min (Figure 3).

**Figure 4:**
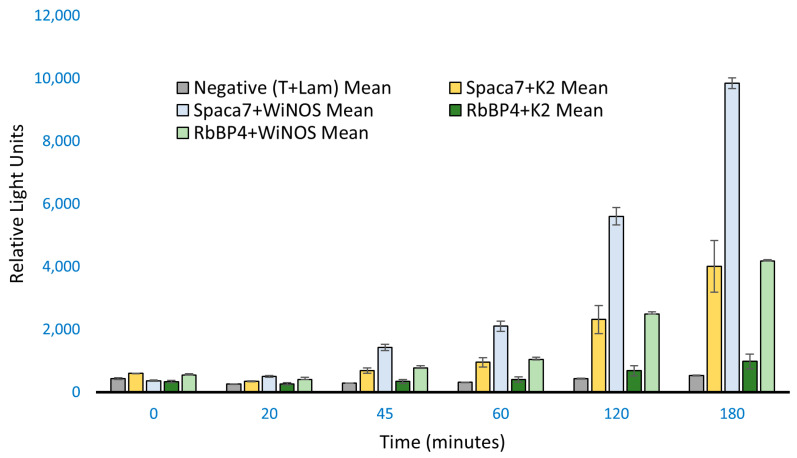
ProLabel assay with full-length iNOS as bait. K2 is the iNOS peptide fragment as used before compared to the full-length iNOS, as shown by WiNOS. Here, the incubation time was increased to 180 min. The Spaca7 and RbBP4 show stronger signals.

So far, we have used a specific fragment of iNOS as bait in our study to observe protein–protein interaction with human SPACA7 and RbBP4; however, the whole iNOS (WiNOS) was necessary to be analyzed to determine whether they also work as wildtype/full-length. For this, a new bait (WiNOS) containing a 3.4 kb sequence of iNOS cDNA was amplified and cloned into the mammalian vector employing the same TaKaRa Clontech, Inc.’s In-Fusion cloning strategy. We followed the co-transfection and Co-IP protocols essentially as described earlier. The result (Figure 4) shows that full-length iNOS interacts more strongly with SPACA7 and RbBP4 in HEK293 cells. This indicates that the prey-iNOS binding is more favored when the iNOS is in its entire native state. To support our data, we performed RT-PCR analysis to determine the relative gene expression of whole iNOS, SPACA7, and RbBP4 in the co-transfected cells.

### 3.4. Fluorescence Imaging of Co-Transfected HEK293 Cells Shows Co-Localization of iNOS and Its Interacting Partner Proteins

**Figure 5:**
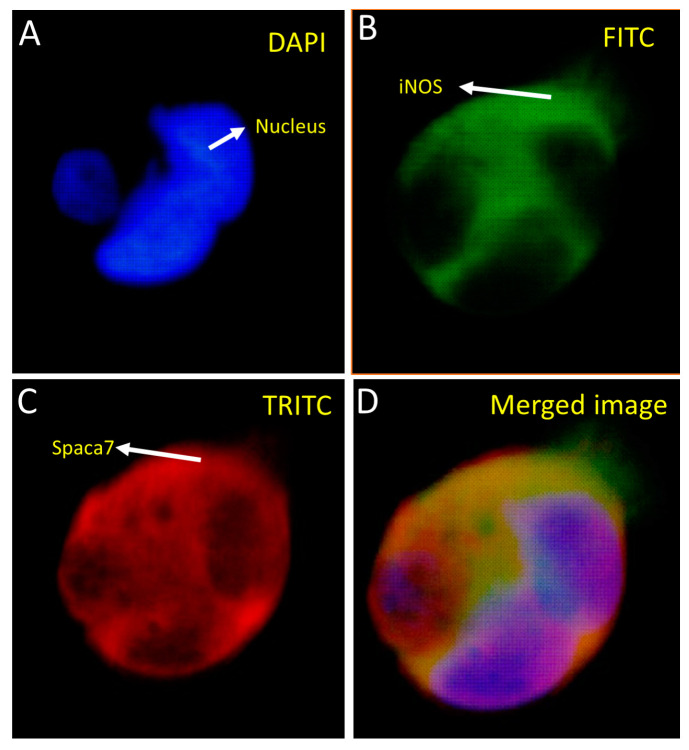
Co-localization of SPACA7 and iNOS in HEK293 cells transfected with pProLabel-SPACA7 and pAcGFP-WiNOS. The nucleus appears blue under the DAPI filter due to the DAPI staining of the nuclear DNA (**A**). The iNOS appears green due to the GFP under the FITC filter (**B**). The SPACA7 appears red under the TRITC filter because of using red-fluorescent secondary antibodies specific to the primary SPACA7 antibody (**C**). Merged image of (**B**,**C**) (**D**). (Magnification 40×).

**Figure 6:**
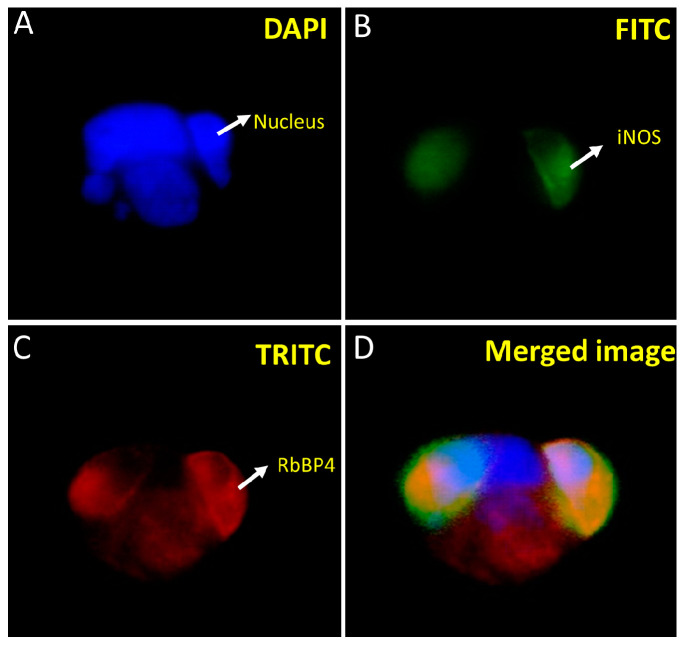
Co-localization of RbBP4 and iNOS in HEK293 cells transfected with pProLabel-RbBP4 and pAcGFP-WiNOS. The nucleus appears blue under the DAPI filter due to the DAPI staining of the nuclear DNA (**A**). The iNOS appears green due to the GFP under the FITC filter (**B**). The RbBP4 appears red under the TRITC filter because of using red-fluorescent secondary antibodies specific to the primary SPACA7 antibody (**C**). Merged image of (**B**,**C**) (**D**). (Magnification 40×).

We carried out co-localization studies of the bait and prey proteins concerning each other. In this regard, the HEK293 cells were stably co-transfected with pAcGFP-WiNOS and pProLabel prey proteins and subjected to immunofluorescence studies by employing an FITC filter to detect GFP-fused bait and a TRITC filter to detect fluorescently labeled secondary antibodies against anti-prey primary antibodies (Figure 5 and Figure 6). The transfection was performed using the CalPhos™ Mammalian Transfection Kit^®^ and transfected cells were seeded on coverslips and placed on the 6-well culture plate. Following the selection of cells under 200 µg/mL of G418, the coverslips were studied by immunofluorescence using primary antibodies specific to the target proteins and fluorescently labeled secondary antibodies. The coverslips were imaged using a fluorescence microscope, as described earlier. The images show that both iNOS and SPACA7 were predominantly localized in the cytosolic regions (Figure 5). Further, on another slide, we looked for co-localization of iNOS and RbBP4 that also exhibited cytosolic localization (Figure 6).

## 4. Discussion

Nitric oxide is a gaseous molecule with diverse physiological functions [23]. However, excessive NO produced by iNOS is thought to be responsible for various diseases, including inflammation-based male infertility [3] and peroxidative damage to renal tubules [7]. One study found a link between NO concentration and duration in that optimum NO concentration affects sperm capacitation [24].

Sustained NO levels can target multiple proteins and modify their functions through S-nitrosylation of the cysteine residues [25]. Despite this, the molecular mechanism underlying NO’s role in male infertility remains unclear. We have been investigating the regulation of NO synthesis in the cell, specifically in the human testis. Our approach was to identify proteins interacting with the iNOS representing amino acids 181–335 and, potentially, to modify their function, thereby regulating NO synthesis. This unique iNOS bait was not previously employed to identify iNOS partner proteins. In this regard, we first showed by Western blotting analysis that the bait protein is expressed in yeast (Figure 2). We also verified any bait-mediated autoactivation of the reporter gene expression in the yeast and whether there was any toxicity in cell growth.

To validate the screening results from the Y2H assay, we sought to verify the iNOS and candidate protein interactions through a co-immunoprecipitation method by employing HEK293 cell lines. In this regard, the same iNOS sequence was cloned into the pAcGFP1-C shuttle vector to express AcGFP1-tagged fusion protein—the AcGFP1 tag functions as the epitope for immunoprecipitation. The prey cDNA containing plasmid vectors from the Y2H assay was also isolated and the individual cDNA was cloned into mammalian pProLabel plasmid vectors. The expression of the bait in fusion with the *GFP* gene was determined by observing fluorescence under the FITC filter of a Nikon Eclipse 80i microscope. For prey proteins, identification was performed through immunostaining. For the ProLabel assay, as the positive control, we included interaction between P53 and SV40 T antigens, which is a well-established interaction [26]. The p53–tag interaction, giving high RLU values, was expected. While the positive gave high values, the negative, on the other hand, showed very significantly low RLU values as samples included ProLabel-SV40 T antigen and AcGFP1-Lam, which are known not to interact. However, out of the five samples, two showed relatively high RLU values, signifying a positive interaction of these two preys with the iNOS bait: (a) the human sperm acrosome-associated protein-7 (SPACA7) and (b) the retinoblastoma-binding protein-4 (RbBP4), respectively.

Fluorescence imaging showed that both the whole iNOS and Spaca7 proteins were localized in the cytosol (Figure 5). Similarly, iNOS and RbBP4 also exhibited cytosolic localization (Figure 6). However, RbBP4 is known to be a nuclear protein [27] and further elucidation of this interaction is required regarding how these two proteins find each other in vivo. One possibility could be that the ProLabel tag fused to the RbBP4 may have misdirected the protein into the transfected cells. To verify this possibility, we inserted the prey protein-coding sequence in pAmCyan1-C1 plasmids that would result in the expression of proteins tagged as CFP (cyan fluorescent protein). The expression of both the bait and prey proteins in the HEK293 cells can be imaged using an E80i Nikon fluorescence microscope under the FITC filter, as both GFP and CFP can be viewed at 515/30 nm. RbBP4 did show nuclear localization after subcloning in pAmCyan1-C1 plasmids emphasizing that this protein is a nuclear protein, as reported before.

The human testis cDNA library screening by performing the Y2H assay resulted in approximately 300 positive clones. The screening process was based on the four reporter gene expressions of the hybrid yeast cells. It is to be noted that this portion of the iNOS contains the crucial amino acid residues for substrate binding and subunit interaction and it is adjacent to the enzyme’s catalytic site [20]. BLAST^®^ analysis of the putative interacting proteins’ respective cDNA samples yielded several interesting proteins and their isoforms, including those involved in sperm motility and sperm membrane association. Further analysis of the same using a Matchmaker^®^ Chemiluminescent Co-IP Assay Kit II^®^ revealed significant positive interactions of iNOS with SPACA7 and RbBP4 separately. These findings represent the first demonstration of such interactions.

SPACA7 is a newly discovered protein in rats. It belongs to a group of acrosome-associated proteins, meaning they are localized in the acrosome, a structure found on the head of sperm. The human counterpart of the *SPACA7* gene is located on chromosome 13 (13q34) and produces a 195 amino acid protein. However, the role of SPACA isoforms in fertility is still unclear [28]. SPACA7 is exclusively expressed in the testis compared to other SPACA isoforms, and the acrosome, where it is found, plays a crucial role in the process of egg–sperm fusion, known as the acrosome reaction. The acrosome contains many essential proteins and enzymes related to spermatogenesis.

**Figure 7:**
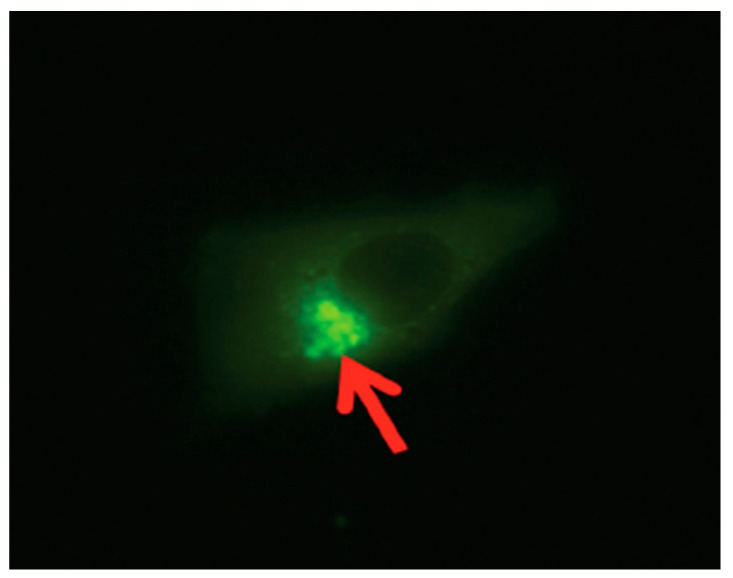
The SPACA7-WiNOS stable cell line under a fluorescent microscope (FITC Filter, 40×).

Studies have shown that SPACA7 distribution in the testis in sequence with the biosynthesis of acrosome in the sperm cell is localized to the Golgi apparatus in the early stages; however, in the later stages of acrosome formation, the SPACA7 was found bound to the acrosome itself [28,29]. Also, iNOS, though initially expressed in the cytosolic region of the cell, eventually is localized to the perinuclear region, more predominantly in the Golgi region of the cell [12]. Interestingly, as in the study mentioned above, we also found a similar finding when we analyzed a stable SPACA7-WiNOS co-transfected cell line, where we discovered that iNOS was more concentrated in the perinuclear region (Figure 7).

**Figure 8:**
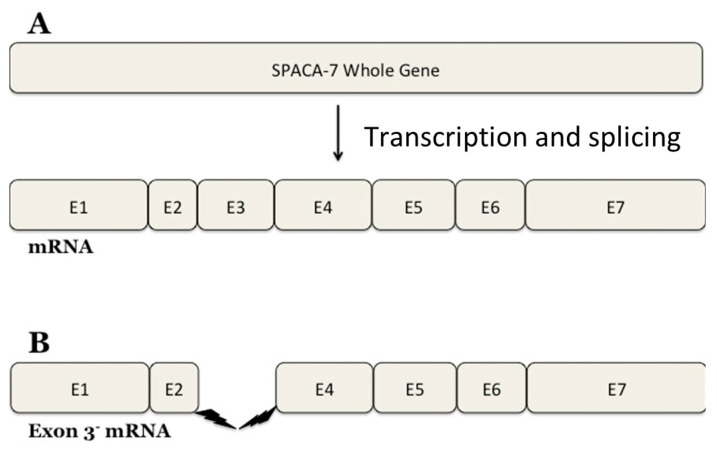
Sequencing of the Spaca7 cDNA revealed a spliced variant. Original spliced mRNA of human SPACA7 (**A**); spliced variant SPACA7 that we found (**B**).

A large quantity of NO is induced by a stimulus when the inducible form of NOS is present [23]. Studies have indicated that iNOS is involved in sperm–oocyte fusion and acrosome reactions [30]. Interestingly, our research has identified a SPACA7 splice variant as deduced from the DNA sequencing data (Figure 8). The *SPACA7* gene comprises seven exons and the expressed protein has six crucial motifs. Our research identified a new splice variant of SPACA7 that has yet to be experimentally observed. Exon 3, encompassing nucleotide bases 291 to 398 of the SPACA7 open reading frame, is missing in this splice variant. The 4Fe-4S ferredoxin-type iron–sulfur binding region of the full-length SPACA7 sequence is located within this region. This domain is absent in the variant SPACA7 protein, so it may be a source of impaired electron transport within the cell. It is still being determined why this specific variant of SPACA7 is showing an interaction with iNOS. We propose that this SPACA7 variant may be produced under unknown physiological conditions in the Golgi during the initial stages of acrosome formation. Here, it possibly interacts with iNOS and is, thus, transported together to the acrosome, where it may regulate the sperm–acrosome reaction.

We have made an unexpected finding of another protein, the retinoblastoma tumor-suppressor binding protein, RbBP4, showing interaction in our assay. This protein is overexpressed in various cancerous cells. It attaches to the tumor-suppressor protein pRb, crucial in regulating the cell cycle and repairing DNA through multiple modifications such as acetylation, methylation, phosphorylation, SUMOylation, and ubiquitination [31]. The inactive form of pRb is commonly found in various cancerous cells [32], and since pRb is not limited to testis cells, it is essential to study RbBP4 in detail. Our finding may open new avenues of research to investigate the potential role of RbBP4–iNOS interaction in either tumor progression or its regression.

## Data Availability

The data presented in this study are available on request from the corresponding author in accordance with the state regulations and appropriate laws.
